# Evaluating risk factors for lung cancer among never-smoking individuals using two Australian studies

**DOI:** 10.1007/s00432-022-04043-9

**Published:** 2022-05-27

**Authors:** Elvin S. Cheng, Marianne F. Weber, Julia Steinberg, Karen Canfell, Xue Qin Yu

**Affiliations:** 1 Sydney School of Public Health, The University of Sydney, Sydney, NSW Australia; 2 Daffodil Centre, The University of Sydney, a joint venture with Cancer Council New South Wales, Sydney, NSW Australia; 3 Prince of Wales Clinical School, University of New South Wales, Sydney, NSW Australia

**Keywords:** Lung cancer, Never-smoking, Risk factor, Asia, Australia

## Abstract

**Purpose:**

Lung cancer (LC) in never-smoking individuals would rank as Australia’s eighth most deadly cancer, yet risk factors remain uncertain. We investigated demographic, lifestyle and health-related exposures for LC among never-smoking Australians.

**Methods:**

Using the prospective 45 and Up Study with 267,153 New South Wales (NSW) residents aged ≥ 45 years at recruitment (2006–2009), we quantified the relationship of 20 potential exposures with LC among cancer-free participants at baseline who self-reported never smoking. Adjusted hazard ratios (HR) and 95% confidence intervals (CI) for incident LC were estimated using Cox regression. The NSW Cancer, Lifestyle and Evaluation of Risk (CLEAR) Study, a case–control study including 10,781 NSW residents aged ≥ 18 years (2006–2014), was used to examine 16 potential LC exposures among cancer-free never-smoking participants. Adjusted odds ratios (OR) and 95% CI of LC were estimated using logistic regression.

**Results:**

There were 226 LC cases among 132,354 cancer-free 45 and Up Study participants who reported never smoking, with a median follow-up of 5.41 years. The CLEAR Study had 58 LC cases and 1316 cancer-free controls who had never smoked. Analyses of both datasets showed that Asian-born participants had a higher risk of LC than those born elsewhere: cohort, adjusted HR = 2.83 (95% CI 1.64–4.89) and case–control, adjusted OR = 3.78 (1.19–12.05). No significant association with LC was found for other exposures.

**Conclusion:**

Our findings support the growing evidence that never-smoking, Asian-born individuals are at higher risk of developing LC than those born elsewhere. Ethnicity could be considered when assessing potential LC risk among never-smoking individuals.

**Supplementary Information:**

The online version contains supplementary material available at 10.1007/s00432-022-04043-9.

## Introduction

Lung cancer (LC) is the leading cause of cancer mortality globally in both sexes combined (Sung et al. 2021). In Australia, it is the fifth most commonly diagnosed cancer and the leading cause of cancer mortality, accounting for 18.1% (9034 deaths) of all cancer deaths in 2019 (Australian Institute of Health and Welfare 2019). Although tobacco smoking is responsible for the majority of LC cases, 15–25% of global cases occur in never-smoking individuals (Ferlay et al. 2018; Sun et al. 2007), defined as those who have smoked fewer than 100 cigarettes in their lifetime (Mak et al. 2016). It is estimated that of LC cases diagnosed in Australia between 2017 and 2026, 16.1% of cases in men and 28.9% in women would not be attributable to active smoking (Laaksonen et al. 2018). LC in individuals that have never smoked has been considered as a separate disease entity from LC that develops in individuals with a smoking history, as they have notable differences in epidemiological, clinicopathologic and molecular characteristics (Couraud et al. 2012; Subramanian and Govindan 2010; Sun et al. 2007; Toh et al. 2006). When compared with lung cancer in individuals with a smoking history, LC among those without a smoking history occurs more frequently among women, individuals of Asian descent and younger age groups, and is predominantly adenocarcinoma in histology type (Antony et al. 2010; Clement-Duchene et al. 2010; Couraud et al. 2012; Okazaki et al. 2016; Sun et al. 2015). When considered as a distinct entity, LC in never-smoking individuals was estimated to be the seventh leading cause of cancer death worldwide in 2000 (Parkin et al. 2005; Sun et al. 2007; Thun et al. 2006), and the eighth leading cause of cancer death in Australia in 2017 (Australian Institute of Health and Welfare 2017; Laaksonen et al. 2018). Despite a growing body of research on LC in never-smoking individuals, there is limited understanding of its aetiology.

The International Agency for Research on Cancer (IARC) Working Group classified radon, passive smoking, various occupational exposures (e.g., asbestos), outdoor air pollution and coal from household combustion as factors other than smoking that can cause LC (Cogliano et al. 2011). However, a significant portion of never-smoking patients with LC have not been exposed to these carcinogens, suggesting that other risk factors remain to be established (Samet et al. 2009).

In this study, we used two Australian studies (a large-scale prospective cohort and a cancer case–control study) with a large sample of never-smoking participants to examine the association of LC with multiple known and potential risk factors that have been studied in other populations: including Asian country of birth (Sun et al. 2007), family history of lung cancer (Cote et al. 2012; Matakidou et al. 2005), passive smoking (Boffetta et al. 2000; Li et al. 2018; Ni et al. 2018; Taylor et al. 2007), chronic obstructive pulmonary disease (COPD) (Gardner et al. 2018), asthma requiring treatment (Pirie et al. 2016), diabetes (Lee et al. 2013), height (Wang et al. 2017), body mass index (BMI) (Zhu and Zhang 2018), physical activity (Zhong et al. 2016), alcohol consumption (Bagnardi et al. 2011), red meat and processed meat intake (Gnagnarella et al. 2018), fruit and vegetable intake (Vieira et al. 2016), anti-hypertensive medications (Hicks et al. 2018; Rotshild et al. 2018; Sipahi et al. 2010) and variables related to female reproductive factors, including use of oral contraceptive pills, age at birth of first child, parity, menopausal age and menopausal hormone therapy (MHT) (Ben Khedher et al. 2017; Stucker et al. 2017).

## Materials and methods

### Study design and populations

Both the 45 and Up Study (Sax Institute, Australia) and the New South Wales (NSW) Cancer, Lifestyle and Evaluation of Risk (CLEAR) Study (Cancer Council NSW) were used for analysis. These two separate studies, one prospective and one retrospective in design, were established over a similar period of time, with largely aligned study questionnaires (taking account of study design differences), and were sampled from the same population (NSW).

The 45 and Up Study is the largest population-based Australian prospective cohort study to date with 267,153 participants at recruitment and data for 266,705 participants available for analysis at the time of data linkage. The study methods have been described in detail elsewhere (Banks et al. 2008; Mealing et al. 2010). In summary, participants were randomly sampled from the Services Australia’s (formerly the Australian Government Department of Human Services) Medicare enrolment database which contains records for all Australian citizens and permanent residents, and some temporary residents and refugees, recruited from February 2006 to December 2009. All NSW residents aged 45 years or over at time of recruitment were eligible to be sampled. Participants joined the study by completing a health and lifestyle questionnaire (available at https://www.saxinstitute.org.au/our-work/45-up-study/questionnaires) and giving informed consent for follow-up through routine data linkage to population-based health databases. For the purpose of this analysis, we used the 45 and Up baseline data linked to cancer notifications from the NSW Cancer Registry (NSWCR; January 1994 to December 2013), hospital records from the Admitted Patients Data Collection (APDC; July 2001 to December 2009), and death notifications from the NSW Registry of Births Deaths & Marriages (February 2006 to December 2013), via probabilistic linkage conducted by the NSW Centre for Health Record Linkage (CHeReL) http://www.cherel.org.au. Data on claims for prescription medications were obtained from the Pharmaceutical Benefits Scheme (PBS; June 2004 to December 2009) supplied by Services Australia, which were linked to the 45 and Up Study baseline data (with deterministic matching) by the Sax Institute using a unique identifier. The study sample was restricted to participants who responded ‘No’ to the question ‘Have you ever been a regular smoker?’ in the baseline questionnaire and did not have conflicting responses to other smoking-related questionnaire items. Never-smoking participants who self-reported a prior history of cancer (other than non-melanocytic skin cancer) or those with a cancer diagnosis recorded in NSWCR between 1994 and baseline were also excluded.

The NSW Cancer, Lifestyle and Evaluation of Risk (CLEAR) Study is an ‘all cancer case-spouse control’ study in which 10,781 participants (45.5% males) were recruited, with 8,550 cases and 2231 controls (Sitas et al. 2015). Participants were NSW residents aged ≥ 18 years recruited between 2006 and 2014. Cases had a self-reported cancer diagnosis within 18 months before enrolment and were identified using both a ‘targeted’ and a ‘non-targeted’ recruitment approach. About 75% of the cases were identified by the ‘targeted’ approach, which used a medical or health related database to identify potential cases via: (i) Sydney South East Clinical Cancer Registry, (ii) Sydney South West Clinical Cancer Registry, (iii) Melanoma Institute of Australia, or (iv) Hospitals Contribution Fund. About 25% of the cases were recruited by a ‘non-targeted’ approach in which the participants responded to community promotion, and face-to-face recruitment at oncology clinics in NSW. Controls were spouses of cases and were self-reported cancer-free (except for self-reported non-melanocytic skin cancer) at the time of recruitment (Sitas et al. 2015). All participants completed a self-administered paper-based health and lifestyle questionnaire after signing the consent form. For our analysis, the study sample was made up of participants who responded ‘No’ to the question ‘Have you ever been a regular smoker?’, with exclusion of those who reported prior history of cancer.

The conduct of the 45 and Up Study was approved by the University of NSW Human Research Ethics Committee. The CLEAR Study was approved by St. Vincent’s Hospital Human Research Ethics Committee. The NSW Population and Health Services Research Ethics Committee approved the record linkage and analysis of the 45 and Up Study data (HREC/14/CIPHS/54) and the analysis of the CLEAR Study data (HREC/14/CIPHS/36).

### Ascertainment of outcomes

In both analyses, the primary outcome was incident LC. In the 45 and Up Study, incident primary cases of LC (classified as C34 in the 10th version of International Statistical Classification of Diseases—Australian modification, ICD10-AM), date of diagnosis and histological sub-type were captured in the NSWCR between the date of recruitment and December 2013. Cancer notification has been a statutory requirement in NSW since 1972 (Tracey et al. 2005). For the CLEAR Study, LC and date of diagnosis were self-reported at recruitment and information on histological sub-type was not available.

### Ascertainment of exposures and covariates

All exposures and covariates of interest are listed in Tables 1 and 2, and are detailed in Supplementary Table 1. Exposures that were self-reported in the baseline questionnaire of both studies included country of birth, family history of LC, current passive smoking, asthma requiring treatment, diabetes, height, BMI, physical activity, alcohol consumption, red meat intake, fruit and vegetable intake, and variables related to female reproductive factors, including oral contraceptive use, MHT, age at birth of first child and parity. Of the variables ascertained from both studies, country of birth, education, height, parity, and age at birth of first child were ascertained using identical questionnaire items. All other variables were ascertained using similar, but slightly different items (see Supplementary Table 1); notably, due to the retrospective design, the CLEAR Study questions mostly collected information about exposures within the last 18 months before participants’ or their spouses’ cancer diagnosis. Moreover, in the 45 and Up Study, the question on passive smoking was limited to current exposure at baseline, and past exposure could not be assessed. There were also additional exposures in the 45 and Up Study including processed meat intake, anti-hypertensive medications, and menopausal age. Prevalent cases of COPD were ascertained from diagnosis codes in the APDC from July 2001 to December 2009 (i.e., for bronchitis, chronic bronchitis, emphysema or COPD; ICD10-AM J40-J44) and/or PBS records from June 2004 to December 2009 (prescription for tiotropium bromide monohydrate, a mainstay treatment for COPD, claimed prior to a participant’s baseline survey date) (Weber et al. 2017). We then categorised country of birth into two groups: ‘Asia’ and ‘non-Asia’ based on the Australian Bureau of Statistics (ABS) Standard Australian Classification of Countries, and all the Asian countries (including India) defined in this study are listed in Supplementary Table 2.

**Table 1:** Distribution of socio-demographic, environmental, lifestyle and other health-related factors for never-smoking individuals at baseline in the 45 and Up Study (2006–2009) and the NSW CLEAR Study* (2006–2014)

Characteristics	45 and up study, *n* = 132,354	CLEAR study, *n* = 1374
Mean age at baseline (SD), years	62.1 (11.1)	59.4 (12.2)
Mean height (SD), cm	167.0 (12.2)	168.1 (11.4)
Socio-demographic factors, *n* (%)		
Women	80,478 (60.8)	802 (58.4)
SEIFA in most disadvantaged quintile	24,117 (18.2)	175 (12.7)
Resided in major cities	71,194 (53.8)	797 (58.0)
Education of University or higher level	35,116 (26.5)	456 (33.2)
Asian born	6516 (4.9)	53 (3.9)
Environmental factors, *n* (%)		
No exposure to passive smoking (home and other)	85,861 (64.9)	1193 (86.8)
Health-related factors, *n* (%)		
Family history lung cancer	12,899 (9.8)	148 (10.8)
Asthma	5657 (4.3)	200 (14.6)
Diabetes	10,607 (8.0)	123 (9.0)
Chronic obstructive pulmonary disease	1980 (1.5)	–
Lifestyle factors, *n* (%)		
Normal body mass index (18.5–2 5.0 kg/m^2^)	47,829 (36.1)	501 (36.5)
Physical activity: 0 session per week	6728 (5.1)	64 (4.7)
Alcohol consumption: > 7 drinks per week	30,574 (23.1)	326 (23.7)
Fruit intake: 0 serve per day	6568 (5.0)	115 (8.4)
Vegetable intake: 0 serve per day	1195 (0.9)	< 5
Red meat intake: > 5 times per week	12,637 (9.6)	90 (6.6)
Processed meat intake: > 2 times per week	18,095 (13.7)	–
Female hormonal and reproductive factors ^§^, *n* (%)		
Nulliparous	8132 (10.1)	84 (10.5)
Age at first child birth ≥ 30 years old	10,534 (13.1)	109 (13.6)
Use of hormonal contraceptives: Ever	53,008 (65.9)	674 (84.0)
Use of hormone replacement therapy: Ever	27,825 (34.6)	308 (38.4)
Menopausal age ≥ 50 years old	30,095 (37.4)	–

^§^For women only

*New South Wales Cancer Lifestyle and Evaluation of Risk Study

**Table 2:** Risk of lung cancer among never-smoking individuals in the 45 and Up Study and the NSW CLEAR Study in relation to demographic, health and lifestyle factors

45 and up study						CLEAR study					
Exposures	Lung cancer			Fully adjusted model*		Exposures	Lung cancer			Fully adjusted model^	
	Yes (%)	No (%)	*p* value	HR (95% CI)	*p* value		Case (%)	Control (%)	*p* value	OR (95% CI)	*p* value
	*n* = 226	*n* = 132,128					*n* = 58	*n* = 1316			
Mean age (SD), years	72.4 (11.0)	62.1 (11.1)	< 0.0001			Mean age (SD), years	64.2 (10.8)	59.2 (12.3)	0.0012		
Mean height (SD), cm	165.9 (10.3)	167.5 (10.1)	0.026	1.01 (0.98, 1.03)	0.67	Mean Height (SD), cm	165.2 (11.1)	168.2 (11.4)	0.062	0.44 (0.02, 9.64)	0.60
Sex			0.83		0.88	Sex			0.094		0.71
Male	87 (38.5)	51,789 (39.2)		1.00 (Reference)		Male	18 (31.0)	554 (42.1)		1.00 (Reference)	
Female	139 (61.5)	80,339 (60.8)		1.03 (0.67, 1.60)		Female	40 (69.0)	762 (57.9)		1.16 (0.53, 2.53)	
Country of birth			0.0063		0.0002	Country of birth			0.0088		0.025
Non-Asian	206 (91.1)	125,632 (95.1)		1.00 (Reference)		Non-Asian	52 (89.7)	1269 (96.4)		1.00 (Reference)	
Asian	20 (8.9)	6,496 (4.9)		2.83 (1.64, 4.89)		Asian	6 (10.3)	47 (3.6)		3.78 (1.19, 12.05)	
Passive smoking (hours/week)			0.34		0.37	Passive smoking exposure			0.34		0.65
None	141 (62.4)	85,720 (64.9)		1.00 (Reference)		None	> 47	1145 (87.0)		1.00 (Reference)	
≤ 3.5	32 (14.2)	21,738 (16.4)		1.12 (0.75, 1.68)		Not under 13	< 5	44 (3.3)		2.28 (0.61, 8.57)	
> 3.5	11 (4.9)	10,375 (7.9)		0.67 (0.35, 1.28)		Yes under 13	< 5	91 (6.9)		1.20 (0.40, 3.61)	
Missing	42 (18.6)	14,295 (10.8)				Missing	0	36 (2.7)			
Physical activity (min/week)			0.19		0.49	Physical activity (intensity level)			0.48		0.48
Nil	17 (7.5)	6711 (5.1)		1.00 (Reference)		Nil to very low activity	27 (46.6)	513 (39.0)		1.00 (Reference)	
0–150	38 (16.8)	21,370 (16.2)		1.06 (0.53, 2.12)		Low activity	> 9	218 (16.6)		1.54 (0.72, 3.30)	
150–300	28 (12.4)	20,935 (15.8)		0.75 (0.35, 1.59)		Moderate activity	17 (29.3)	473 (35.9)		0.84 (0.41, 1.71)	
≥ 300	128 (56.6)	79,488 (60.2)		1.10 (0.58, 2.10)		High activity	< 5	76 (5.8)		0.31 (0.04, 2.51)	
Missing	15 (6.6)	3624 (2.7)									
BMI (kg/m^2^)			0.0006		0.052	BMI before dx (kg/m^2^)			0.68		0.86
< 18.5	37 (16.4)	11,917 (9.0)		1.98 (1.16, 3.39)		< 18.5	8 (13.8)	145 (11.0)		1.58 (0.18, 13.76)	
≥ 18.5 to < 25	85 (37.6)	47,744 (36.1)		1.00 (Reference)		≥ 18.5 to < 25	24 (41.4)	477 (36.3)		1.00 (Reference)	
≥ 25 to < 30	72 (31.9)	47,224 (35.7)		0.92 (0.65, 1.30)		≥ 25 to < 30	18 (31.0)	461(35.0)		0.82 (0.42, 1.59)	
≥ 30	32 (14.2)	25,243 (19.1)		1.01 (0.64, 1.59)		≥ 30	8 (13.8)	233 (17.7)		0.77 (0.31, 1.89)	
Alcohol consumption (drinks/week)			0.026		0.94	Alcohol consumption (drinks/week)			0.23		0.69
≥ 0 to < 1	106 (46.3)	49,383 (37.3)		1.07 (0.67, 1.70)		≥ 0 to < 1	25 (43.1)	459 (34.9)		0.96 (0.41, 2.26)	
≥ 1 to ≤ 3.5	30 (13.1)	22,992 (17.4)		1.00 (Reference)		≥ 1 to ≤ 3.5	9 (15.5)	241 (18.3)		1.00 (Reference)	
> 3.5 to ≤ 7	40 (17.5)	26,336 (19.9)		0.94 (0.54, 1.63)		> 3.5to ≤ 7	15 (25.9)	285 (21.7)		1.11 (0.45, 2.75)	
> 7	45 (19.7)	30,601 (23.1)		1.16 (0.69, 1.96)		> 7	8 (13.8)	318 (24.2)		0.58 (0.20, 1.64)	
Missing	8 (3.5)	3049 (2.3)				Missing	1 (1.7)	13 (1.0)			
Fruit intake (serves/day)			0.076		0.59	Evening fruit intake (times/week)			0.28		0.26
≥ 0 to < 1	7 (3.1)	6585 (5.0)		1.00 (Reference)		≥ 0 to < 7	21 (36.2)	593 (45.1)		1.00 (Reference)	
≥ 1 to < 2	55 (24.3)	40,454 (30.6)		0.99 (0.42, 2.33)		≥ 7	30 (51.7)	619 (47.0)		1.39 (0.72, 2.68)	
≥ 2	146 (64.6)	79,424 (60.1)		1.19 (0.52,2.73)		Missing	7 (12.7)	104 (7.9)			
Missing	18 (8.0)	5665 (4.3)									
Vegetable intake (serves/day)			0.25		0.45	Evening vegetable intake (times/week)			0.94		0.24
≥ 0 to < 3	47 (20.8)	36,024 (27.3)		1.00 (Reference)		≥ 0 to < 7	22 (37.9)	492 (37.4)		1.00 (Reference)	
≥ 3 to < 5	63 (27.9)	37,271 (28.2)		1.29 (0.85, 1.97)		≥ 7	34 (58.6)	777 (59.0)		0.66 (0.35, 1.26)	
≥ 5	72 (31.9)	41,133 (31.1)		1.08 (0.70, 1.67)		Missing	2 (3.5)	47 (3.6)			
Missing	44 (19.5)	17,700 (13.4)									
Red meat intake (times/week)			0.045		0.57	Evening red meat intake (times/week)			0.14		0.13
Never	31 (13.7)	14,373 (10.9)		1.00 (Reference)		Never	1 (1.7)	24 (1.8)		1.00 (Reference)	
> 0 to ≤ 2	52 (23.0)	35,869 (27.2)		0.89 (0.52, 1.50)		> 0 to ≤ 2	16 (27.6)	421 (32.0)		0.70 (0.08, 6.00)	
> 2 to ≤ 5	102 (45.1)	64,977 (49.2)		0.94 (0.58, 1.54)		> 2 to ≤ 5	30 (51.7)	741 (56.3)		0.80 (0.10, 6.64)	
> 5	31 (13.7)	12,606 (9.5)		1.26 (0.69, 2.27)		> 5	8 (13.8)	82 (6.2)		2.41 (0.26, 22.77)	
Missing	10 (4.4)	4,303 (3.3)				Missing	3 (5.2)	48 (3.7)			
Family history of LC			0.37		0.39	Family history of LC			0.45		0.40
No	200 (88.5)	119,255 (90.3)		1.00 (Reference)		No	50 (86.2)	1176 (89.4)		1.00 (Reference)	
Yes	26 (11.5)	12,873 (9.7)		1.22 (0.78, 1.93)		Yes	8 (13.8)	140 (10.6)		1.43 (0.62, 3.26)	
Asthma requiring treatment			0.079		0.33	Asthma requiring treatment			0.55		0.74
No	211 (93.4)	126,486 (95.7)		1.00 (Reference)		No	48 (82.8)	1126 (85.6)		1.00 (Reference)	
Yes	15 (6.6)	5642 (4.3)		1.35 (0.73, 2.50)		Yes	10 (17.2)	190 (14.4)		0.85 (0.34, 2.16)	
Diabetes			0.48		0.87	Diabetes			0.58		0.49
No	205 (90.7)	121,539 (92.0)		1.00 (Reference)		No	> 53	1197 (91.0)		1.00 (Reference)	
Yes	21 (9.3)	10,586 (8.0)		0.87 (0.52, 1.46)		Yes	< 5	119 (9.0)		0.64 (0.18, 2.27)	
Processed meat intake (times/week)			0.085		0.12						
Never	59 (26.1)	30,430 (23.0)		1.00 (Reference)							
> 0 to ≤ 1	54 (23.9)	43,752 (33.1)		0.61 (0.39, 0.96)							
> 1 to ≤ 2	34 (15.0)	20,275 (15.4)		0.99 (0.60, 1.62)							
> 2	24 (10.6)	18,071 (13.7)		0.88 (0.51, 1.50)							
Missing	55 (24.3)	19,600 (14.8)									
COPD			< 0.0001		0.20						
No	215 (95.1)	130,159 (98.5)		1.00 (Reference)							
Yes	11 (4.9)	1,969 (1.5)		1.63 (0.77, 3.44)							
Anti-hypertensive medication (ACEI, ARB, CCB)			0.0018		0.92						
Nil	156 (69.0)	105,344 (79.7)		1.00 (Reference)							
Only ACEI	24 (10.6)	9663 (7.3)		1.24 (0.77, 2.01)							
Only ARB	25 (11.1)	10,974 (8.3)		1.06 (0.65, 1.73)							
Only CCB	11 (4.9)	3073 (2.3)		1.36 (0.68, 2.70)							
Any 2 or all of them	10 (4.4)	3070 (2.3)		1.17 (0.54, 2.54)							

*SD* standard deviation, *BMI* body mass index, *LC* lung cancer, *COPD* chronic obstructive pulmonary disease, *ACEI* angiotensin converting enzyme inhibitor, *ARB* angiotensin receptor blocker, *CCB* calcium channel blocker

*Multivariable model with age as the underlying time variable and adjusted for sex, SES, region of residence, educational level, height, Asian country of birth, family history of lung cancer, passive smoking, COPD, asthma requiring treatment, diabetes, BMI, physical activity, alcohol consumption, fruit intake, vegetable intake, red meat intake, processed meat intake and anti-hypertensive medication

^Multivariable model adjusted for age, sex, SES, region of residence, educational level, height, Asian country of birth, family history of lung cancer, passive smoking, asthma requiring treatment, diabetes. BMI, physical activity, alcohol consumption, evening fruit intake, evening vegetable intake and evening red meat intake

Potential confounders included as covariates were age at baseline, sex, SEIFA [Socio-Economic Indexes for Areas based on the Index of Relative Socio-Economic Disadvantage according to participants’ residential address (Australian Bureau of Statistics 2011b)], ARIA (region of residence based on Accessibility/Remoteness Index of Australia) and highest educational level.

### Statistical analysis

For the 45 and Up Study, participants were followed up from baseline to the time of LC diagnosis, or were censored at death or on 31 December 2013, whichever occurred first. We calculated the proportion of LC with each histological subtype. We calculated adjusted hazard ratios (HRs) and 95% confidence intervals (CIs) for association between each exposure and LC using Cox proportional hazards regressions. HRs were estimated with three levels of adjustment: (i) in a basic analysis with attained age as the underlying time variable (Korn et al. 1997); (ii) with additional adjustment for sex, SEIFA, region of residence and education; and (iii) with adjustment for all the covariates (and exposures). The proportional hazards assumption in the Cox regression models was examined using the method by Lin et al. (1993).

For the CLEAR Study, we used logistic regression to estimate adjusted odds ratios (ORs) and 95% CIs for association between each exposure and lung cancer. ORs were estimated with three levels of adjustment, by adjusting for: (i) baseline age; (ii) baseline age, sex, SEIFA, region of residence and education; (iii) all covariates and exposures.

In both studies, multivariable analyses of female reproductive factors were restricted to women, with adjustment for age, height, demographic factors, Asian country of birth, family history of lung cancer, passive smoking, BMI, lifestyle factors, reproductive factors, and COPD (for the 45 and Up Study only). All statistical analyses were performed in SAS version 9.4 and significance was defined as *p* < 0.05.

### Sensitivity analyses

Three sensitivity analyses were performed. In the 45 and Up Study, there was a high proportion (10.8%) of missing values in the variable related to current passive smoking. We performed a sensitivity analysis in which the total duration of current passive smoke exposure (at home and other places) for participants with missing values (*n* = 14,337) was treated as (a) 0 h/week or (b) > 3.5 h/week, to check for the effect of each assumption. Second, to minimise the potential for reverse causality due to weight loss as an early symptom of LC, we excluded data in the first 2 years of follow-up (i.e., to start the follow-up analysis from the third year onwards) in the sensitivity analysis examining the association of BMI with LC. Lastly, as both BMI and COPD have been known to be independent risk factors and yet also in the causal pathway, they were excluded from the full models in this sensitivity analysis to assess their effects to the exposures.

## Results

In the 45 and Up Study cohort, there were 151,984 never-smoking participants (57.0%) and 19,630 participants with a cancer diagnosis prior to baseline were excluded, which resulted in 132,354 participants available for analysis. During a median follow-up of 5.41 years (SD = 1.03; interquartile range 5.26–5.87 years), 226 participants developed LC (139 women and 87 men). The distribution of cases by histological sub-type is presented in Supplementary Table 3. Adenocarcinoma accounted for 121 cases (53.5% of cases, 79 women and 42 men), followed by squamous cell carcinoma (8.4%) and large cell carcinoma (8.4%).

In the CLEAR Study, there were 5,996 never-smoking participants. After excluding 39 controls and 2 cases with self-reported prior history of cancer, and 4581 cases of cancers other than LC, data on 1374 participants were available for analysis: 58 LC cases (40 women and 18 men; median time from diagnosis to enrolment = 179 days) and 1316 controls.

Socio-demographic, health-related and lifestyle characteristics of never-smoking participants in the two studies are compared in Table 1. The two groups had very similar distributions for the variables of Asian country of birth, family history of lung cancer, BMI, alcohol consumption, diabetes and parity, and were broadly comparable in SEIFA, highest educational level and use of MHT. The distribution of passive smoke exposure and physical activity differed between the two studies.

The adjusted HRs for associations between 20 exposures and LC in the 45 and Up Study are shown in Tables 2 and 3. After adjusting for age alone in the basic model, Asian country of birth, BMI and COPD were significantly associated with LC (see Supplementary Table 4). When all covariates (and exposures) were included in the multivariable regression, only ‘Asian country of birth’ was significantly associated with LC (HR = 2.83 for participants born in Asia compared to participants born elsewhere; 95% CI 1.64–4.89). There was no evidence of non-proportionality in the Cox regressions.

**Table 3:** Risk of lung cancer among never-smoking individuals in the 45 and Up Study and the NSW CLEAR Study in relation to female reproductive and hormonal factors

Exposures	45 and up study (*N* = 80,478)					CLEAR study (*N* = 802)				
	Lung cancer					Lung cancer				
	Yes (%)	No (%)	*p* value	Fully adjusted model*		Case (%)	Control (%)		Fully adjusted model^	
	*n* = 139	*n* = 80,339		HR (95% CI)	*p* value	*n* = 40	*n* = 762	*p* value	OR (95% CI)	*p* value
Parity			0.10		0.65			0.11		0.91
0	15 (10.8)	8,117 (10.1)		1.99 (0.53, 7.56)		< 5	84 (11.0)		/	
1	7 (5.0)	6,171 (7.7)		1.00 (Reference)		< 5	55 (7.2)		1.00 (Reference)	
2	37 (26.6)	27,506 (34.2)		1.19 (0.49, 2.92)		14 (35.0)	287 (37.7)		1.47 (0.29, 7.50)	
≥ 3	79 (56.8)	38,145 (47.4)		1.44 (0.60, 3.47)		23 (57.5)	336 (44.1)		1.78 (0.33, 9.56)	
Age at birth of first child, years			0.23		0.60			0.94		0.69
< 25	67 (48.2)	34,789 (43.3)		1.00 (Reference)		22 (55.0)	413 (54.2)		1.00 (Reference)	
≥ 25 to < 30	32 (23.0)	23,222 (28.9)		0.73 (0.45, 1.20)		12 (30.0)	246 (32.3)		0.69 (0.27, 1.72)	
≥ 30	15 (10.8)	10,519 (13.1)		0.88 (0.44, 1.74)		6 (15.0)	103 (13.5)		0.97 (0.29, 3.30)	
Menopausal hormone therapy			0.62		0.73			0.28		0.49
Never user	82 (59.0)	50,195 (62.5)		1.00 (Reference)		25 (62.5)	461 (60.5)		1.00 (Reference)	
Former user	37 (26.6)	20,161 (25.1)		0.82 (0.51, 1.32)		< 15	220 (28.9		0.97 (0.43, 2.19)	
Current user	10 (7.2)	7617 (9.5)		0.90 (0.44, 1.86)		< 5	73 (9.6)		0.18 (0.02, 1.56)	
Hormonal contraceptive use			<0.0001		0.56			0.63		0.63
No	50 (36.0)	18,597 (23.2)		1.00 (Reference)		7 (17.5)	114 (15.0)		1.00 (Reference)	
< 5 years	22 (15.8)	14,437 (18.0)		1.24 (0.67, 2.27)		32 (80.0)	642 (84.3)		1.11 (0.39, 3.17)	
≥ 5 years	42 (30.2)	38,507 (47.9)		0.87 (0.51, 1.49)						
Menopausal age, years			0.44		0.16					
< 45	14 (10.1)	5,879 (7.3)		1.00 (Reference)						
≥ 45 to < 50	23 (16.6)	12,076 (15.0)		1.04 (0.49, 2.23)						
≥ 50	49 (35.3)	30,046 (37.4)		0.74 (0.37, 1.50)						

Numbers of cases for various exposures do not always sum to totals due to missing values

*Multivariable model with age as the underlying time variable and adjusted for SES, region of residence, educational level, height, Asian country of birth, family history of lung cancer, passive smoking, chronic obstructive pulmonary disease, physical activity, body mass index, alcohol consumption, fruit intake, vegetable intake, red meat intake, parity, age at birth of first child, hormonal contraceptive use, hormone replacement therapy and menopausal age

^Multivariable model adjusted for age, SES, region of residence, educational level, height, Asian country of birth, family history of lung cancer, passive smoking, physical activity, body mass index, alcohol consumption, fruit intake, vegetable intake, red meat intake, parity, age at birth of first child, hormonal contraceptive use, hormonal replacement therapy

The adjusted ORs for associations between 16 exposures and incident LC in the CLEAR Study are also shown in Tables 2 and 3. When adjusted for all covariates in the multivariable regression, only ‘Asian country of birth’ was statistically significant (OR = 3.78; 95% CI 1.19–12.05) (see also Supplementary Table 5).

In both studies, no statistically significant association was found between LC and other potential risk factors in the multivariable models: current or early childhood passive smoking, family history of LC, asthma requiring treatment, diabetes, height, physical activity, BMI, alcohol consumption, fruit intake, vegetable intake, red meat intake, anti-hypertensive medications or processed meat intake (the last two applied to the 45 and Up Study only). Also, no significant association was found between female reproductive factors (oral contraceptive use, age at birth of first child, parity, MHT and menopausal age) and LC.

### Sensitivity analysis

Setting missing values for current passive smoking to (a) 0 h/week or (b) > 3.5 h/week in the sensitivity analyses did not change the association with LC in the 45 and Up Study. Fully adjusted HRs for the highest exposure (> 3.5 h/week) compared to no exposure in the sensitivity analyses were: (a) 0.69 (95% CI 0.37–1.32) and (b) 0.87 (95% CI 0.61–1.25), while the HR in the main analysis was 0.67 (95% CI 0.35–1.28). Second, despite a borderline inverse association between BMI and LC in the main analysis (multivariable HR = 1.98 [95% CI 1.16–3.39; *p* = 0.052] for underweight versus normal weight), when excluding data in the first 2 years of follow-up, the association between BMI and LC was not significant (corresponding multivariable HR = 1.66 [95% CI 0.81–3.40; *p* = 0.27]). Lastly, when BMI and COPD were excluded from the full models, the results showed no appreciable differences from the main analysis.

## Discussion

This is the first Australian study to systematically evaluate the association of demographic, lifestyle, and health-related factors with LC in never-smoking individuals. Asian country of birth was the only factor significantly associated with LC, with results from both a cohort and case–control study showing that risk was around three times greater for Asian-born participants compared to those born elsewhere (including Australia). No significant association was found between LC and other ascertainable factors, including passive smoking, family history of lung cancer, asthma requiring treatment, diabetes, height, anti-hypertensive medications, several lifestyle factors and female reproductive factors. Of note, women were over-represented (over 60%) among those diagnosed with LC in both study groups, and in the cohort study, the majority of LC cases were adenocarcinoma (53.5%).

Whilst many epidemiological studies have reported a higher proportion of never-smoking LC patients among all LC cases in Asian countries than in many non-Asian countries (Cho et al. 2017; Sun et al. 2007; Wang et al. 2010; Zhou and Zhou 2018), LC risk associated with Asian-born individuals has rarely been assessed. Our finding provides direct evidence that never-smoking individuals born in Asia are at a significantly higher risk of developing LC compared to those born elsewhere. The higher LC risk in never-smoking individuals born in Asia in Australia could be due to either genetic or environmental factors or both, which can be difficult to disentangle. Some studies have suggested that inherited genetic susceptibility may play an important role in lung carcinogenesis (Gaughan et al. 2013; Matakidou et al. 2005), while others have explained the high proportion of LC in this sub-group as a result of the low smoking prevalence amongst Asian women (Cho et al. 2017; Wang et al. 2010; Zhou and Zhou 2018). As the causal pathways remain to be determined, further studies would need to investigate this. We also note that Asian-born participants were under-represented in both the 45 and Up Study and the CLEAR Study (3.4% and 3.2%, respectively) compared to the NSW population [12.3% of people over 45 years of age according to the 2011 census; (Australian Bureau of Statistics 2011a)]. Thus, we would expect an even higher proportion of all NSW or Australian never-smoking LC cases to be in Asian-born sub-groups of the population (Colditz 2010; Ponsonby et al. 1996).

In previous studies such as the United Kingdom Million Women Study which examined 34 potential risk factors for 1469 LC cases among 634,039 never-smoking participants during 14 years of follow-up (Pirie et al. 2016), ‘non-white vs. white ethnicity’, ‘asthma requiring treatment vs. not’ and taller stature were found to be significantly associated with LC. We did not detect significant associations between LC and other potential risk factors including asthma and height, and it is likely that our analysis was not sufficiently powered to detect a difference for many of these factors.

Likewise, our results did not show any association of LC with family history of LC. While the genetic pathways in lung carcinogenesis remain to be fully ascertained, several epidemiological studies have provided evidence to suggest familial aggregation of LC (Matakidou et al. 2005), for which inherited susceptibility may be partially responsible (Gorlova et al. 2007). To minimise the effects of shared environmental factors such as smoking habits within families, several studies have also examined familial risk of LC among never-smoking individuals. In a meta-analysis of 11 case–control studies which specifically focused on never-smoking participants, a family history of LC in one or more affected relatives was associated with an increased risk of LC (Matakidou et al. 2005). In a pooled analysis of 24 case–control studies with 3,301 never-smoking LC cases, it was found that LC was associated with a family history of LC in a sibling, but not with a history of LC in a parent, thus suggesting, according to the authors, the possible involvement of a recessive gene effect (Cote et al. 2012); although it is difficult to fully account for the complex patterns of passive vs. active smoking exposures over the lifetime of individuals at risk.

Passive smoking, one of the most widely studied risk factors for LC among never-smoking individuals (Couraud et al. 2012), was classified as a cause of LC by the IARC Working Group in 2004 and 2012 (International Agency for Research on Cancer 2004; International Agency for Research on Cancer 2012). However, a more recent meta-analysis of 41 studies did not report a statistically significant summary relative risk between passive smoking and LC for seven cohort studies (Ni et al. 2018). Another more recent meta-analysis of 28 case–control studies assessed the association between long‑term exposures to passive smoking and LC incidence in China and reported that the pooled OR for exposure from parents was higher than that from a spouse or work (Li et al. 2018). This raised concerns about a higher risk of LC in relation to childhood exposure than adulthood exposure. However, at least one meta-analysis did not provide evidence of an increased LC risk for passive smoking exposure in childhood (Boffetta et al. 2000). Our analyses did not show any association of LC with passive smoking, although our cohort study only captured current or recent passive smoke exposure at baseline, with no information about exposure over the life-course. In contrast, studies that reported a significant association between LC and passive smoking were usually based on long term exposures such as living or working with smokers for a period of time, which are less likely to be subject to exposure misclassification or other biases (International Agency for Research on Cancer 2012).

Health behaviours have also been extensively studied as another group of potential exposures related to LC. These factors include physical activity, alcohol consumption, dietary intake of fruits, vegetables, meat, and to a certain extent, BMI. According to the third (2018) report of World Cancer Research Fund/American Institute for Cancer Research (WCRF/AICR), the evidence for a protective effect of physical activity against LC was limited (World Cancer Research Fund/American Institute for Cancer Research 2018). Several epidemiological studies and meta-analyses have reported an inverse association between physical activity and LC among those with a history of smoking, but not among those without (Borch et al. 2019; Brenner et al. 2016; Zhong et al. 2016), which raised the possibility of the residual confounding effect of smoking. Similarly, the inverse association between BMI and LC has been consistently demonstrated in many studies (Sanikini et al. 2018; Smith et al. 2012), but its interpretation remains controversial due to residual confounding by smoking and possible reverse causality bias. In our analysis, the 45 and Up Study showed a borderline inverse association between BMI and LC with a p value of 0.052 in the fully adjusted model, but after excluding cases diagnosed in the first 2 years of follow-up in a sensitivity analysis, the statistical association disappeared (*p* = 0.34). Regarding dietary factors, it has been suggested that dietary intake of certain foods or beverages is associated with LC risk. According to the third report (2018) by the WCRF/AICR (World Cancer Research Fund/American Institute for Cancer Research 2018), there was limited evidence for fruit and vegetable intake being associated with a decrease in risk, limited evidence for red meat and alcohol intake being associated with an increase in risk, and probable evidence for processed meat intake and increased LC risk. The possibility of residual confounding with active or passive smoking and fruit and vegetable intake should be borne in mind. Our results did not demonstrate any association between these factors and LC.

The predominance of women amongst never-smoking LC cases has led to investigation into the potential roles of female reproductive and hormonal factors in LC, and estrogen has been considered to play a significant role in lung carcinogenesis (Hsu et al. 2017). Numerous studies have examined the association between LC and estrogen-dependent factors including parity, age at first live birth, oral contraceptive use, MHT use, menarche age and menopausal age, but the findings have been inconsistent across studies (Ben Khedher et al. 2017; Brinton et al. 2011; Clague et al. 2011; Kreuzer et al. 2003; Liu et al. 2005; Stucker et al. 2017), and our analyses found no association.

Several other factors have also been suggested as being potentially associated with an increased risk of LC. These factors include COPD, possibly due to chronic inflammation (Gardner et al. 2018) or induction of increased levels of oxidative stress (Mateu-Jiménez et al. 2016), and diabetes, with the neoplastic process possibly occurring through the mechanisms of insulin resistance, hyperglycaemia and chronic inflammation (Lee et al. 2013). It has also been suggested that the use of some anti-hypertensive medications could be associated with LC risk. One of the hypothesised neoplastic mechanisms is related to the renin–angiotensin system, whereby angiotensin converting enzyme inhibitors might enhance angiogenesis via vascular endothelial growth factor and thus promote tumour progression (Hicks et al. 2018; Ishihara et al. 2001).

This study has several strengths. Both datasets provide a wide range of identical or similar questionnaire items on health and lifestyle sampled from the same population (NSW residents). The study sample also has a notable proportion of overseas born residents (Australian Bureau of Statistics 2011a), with the advantage of comparing a large number of participants born in different countries. However, there are some limitations too. The lack of association for all but one potential risk factor in our analyses could be due to low statistical power because of the relatively small number of cases. The relatively short follow-up period for the 45 and Up Study (median of 5.41 years) was another limitation, compared to prior studies with follow-up period of > 10 years. Another limitation of our analyses is that smoking status was self-reported in both studies, so there could be some misclassification with some never-smoking participants having a history of former or light smoking, leading to potential residual confounding by active smoking. As previously discussed, passive smoking exposure over the life-course could not be ascertained in our cohort study. Also, some misclassification could have occurred for other exposures due to social desirability bias, such as physical activity and alcohol consumption, which might bias the results towards the null. Moreover, the CLEAR Study has potential for selection bias due to the way cases and controls were recruited, and the data may be affected by survival bias as cases were recruited up to 18 months after diagnosis of lung cancer. Nevertheless, survival for never-smoking lung cancer cases is better than their smoking counterparts (Yu et al. 2022), and > 50% of the cases were recruited within the first 6 months after diagnosis. Also, for the 20% of the participants linked to the population-based NSW Cancer Registry data, 96% were verified by the Registry [which is ‘gold standard’ for identifying incident cancer cases in a given geographical region (Goldsbury et al. 2017; Kemp et al. 2013)], and the positive predictive value of self-reported lung cancer was 94.6% (Sitas et al. 2015). There is also a concern that as the questionnaires of both studies were only available in English, this would have limited the participation of people with insufficient literacy in English (Banks et al. 2008), and reduced the heterogeneity of the study samples. As a result, the populations of the non-English speaking individuals were likely to be under-represented, which may potentially be a limitation for the assessment of Asian country of birth.

## Conclusion

We found in two Australian studies that never-smoking participants born in Asia are at higher risk of developing LC than those born elsewhere. This adds to the evidence base suggesting an association between Asian ethnicity and LC risk among individuals that have never smoked, though it is difficult to disentangle whether the underlying cause is genetic and/or environmental. As timely diagnosis of LC remains a challenge due to atypical and complex presentations of a number of LC patients (Neal et al. 2015), ethnicity could potentially be considered as one of the criteria for early diagnosis or screening interventions of LC in clinical settings. Further research in diverse populations is needed to improve our understanding of the aetiology of LC in the never-smoking population.

## Supplementary Information

Below is the link to the electronic supplementary material.

Supplementary file1 (DOCX 174 kb)

## Data Availability

Due to confidentiality restrictions in the ethics approval of this study, de-identified information may be released. The authors were only allowed to publish results from the data due to confidentiality conditions.
