# Supplementary material for: Evaluating risk factors for lung cancer among never-smoking individuals using two Australian studies
Source: J Cancer Res Clin Oncol. 2022 May 27;148(10):2827–40. doi: 10.1007/s00432-022-04043-9 (PMC9470598; doi:10.1007/s00432-022-04043-9)
Supplement: Supplementary file 1 — Supplementary file1 (DOCX 174 kb) [file 432_2022_4043_MOESM1_ESM.docx]

**Supplementary Information**

**Supplementary Table 1. Summary table for ascertainment of exposures and covariates in the 45 and Up Study and CLEAR Study**

| **Variables** | **Questionnaire items identical for both 45 and Up Study and CLEAR Study** | | | | **Categorical grouping** |
| --- | --- | --- | --- | --- | --- |
| **Age** | *Age at baseline based on*  **Date of Birth and Today’s date** | | | | - **(Continuous variable)** |
| **Country of birth** | **In which country were you born?**  *Where ‘Asia’ comprised ^Ω^: Myanmar, Cambodia, Laos, Thailand, Vietnam, Brunei Darussalam, Indonesia, Malaysia, Philippines, Singapore, Timor-Leste, China, Hong Kong, Macau, Mongolia, Taiwan, Japan, Korea N, Korea S, Bangladesh, Bhutan, India, Maldives, Nepal, Pakistan & Sri Lanka.*  *And ‘Non-Asia’: Australia or elsewhere* | | | | **‘Non-Asia’ *;**  **‘Asia’** |
| **Height** | **How tall are you without shoes (cm)?** | | | | **Men: ‘<175’ *; ‘≥175 to <180’; ‘≥180’ cm**  **Women: ‘<160’ *; ‘≥160 to <165’; ‘≥165’ cm** |
| **Socioeconomic status** | **Socio-Economic Indexes for Area (SEIFA)**  *Based on the Index of Relative Socio-Economic Disadvantage (IRSD) according to participant’s residential address at the baseline survey.* | | | | **Quintiles, with 1* being the most disadvantaged and quintile 5 the least disadvantaged.** |
| **Region of residence** | **Accessibility/Remoteness Index of Australia (ARIA)**  *Based on the participant’s postcode and Accessibility/Remoteness Index of Australia* | | | | **‘Major cities’ *;**  **‘Inner regional’;**  **‘Outer regional/remote/very remote’** |
| **Highest educational level** | **What is the highest qualification you have completed?** | | | | **‘No school certificate or School cert.’ *;**  **‘Higher school or leaving certificate or**  **Trade/apprenticeship or**  **Certificated/diploma’;**  **‘University or higher’** |
| **Age at birth of first child^§^** | **How old were you when you gave birth to your FIRST child?”** | | | | **‘<25’ *; ‘≥25 to <30’; ‘≥30’** |
| **Parity^§^** | **How many children have you given birth to?** | | | | **‘None’; ‘1’ *; ‘2’; ‘3 or more’** |
| **Questionnaire items similar for both 45 and Up Study and CLEAR Study** | | | | | |
| **Variables** | **45 and Up Study** | | **CLEAR Study** | | **Categorical grouping** |
| **Body mass index (BMI)** | *Derived from self-reported height and weight:*  **How tall are you without shoes (cm)? and**  **About how much do you weigh (kg)?** | | *Derived from self-reported height and weight:*  **How tall are you without shoes (cm)? and**  **About how much did you weigh**  **before you, or your partner, became ill?** | | **‘<18.5’;**  **‘≥18.5 to <25’ *;**  **‘≥25 to <30’;**  **‘≥30’ kg/m^2^** |
| **Family history of lung cancer** | **Have your mother, father, brother(s) or sister(s) (blood relatives only) ever had: lung cancer?** | | **Have your mother, father, brother(s), sister(s) (i.e. siblings) or children ever had: lung cancer?** | | **‘Nil or do not know’ *;**  **‘Yes’** |
| **Alcohol consumption** | **About how many alcoholic drinks do you have each week?** | | **Just before you, or your partner, became ill, about how many alcoholic drinks did you have each week?** | | **‘≥0 to <1’;**  **‘≥1 to ≤3.5’*;**  **‘>3.5 to ≤7’;**  **‘>7’ drinks per week** |
| **Self-reported asthma requiring treatment** | **In the last month have you been treated for: asthma?** | | **Before you, or your partner, became ill, were you ever treated for: asthma?** | | **‘No’ *;**  **‘Yes’** |
| **Questionnaire items different for the 45 and Up Study and CLEAR Study** | | | | | |
|  | **45 and Up Study** | | | **CLEAR Study** | |
| **Variables** | **Questionnaire items** | **Categorical grouping** | | **Questionnaire items** | **Categorical grouping** |
| **Passive smoking** | **About how many hours a week are you exposed to someone else’s tobacco smoke (at home, other places)?** | **‘0’ *;**  **‘>0 to ≤3.5’;**  **‘>3.5’ hours per week** | | **(1) Just before you, or your partner, became ill, did you share your home with anyone who smoked?**  **(2) Did your mother smoke when you were a child^?**  **(3) Did your father smoke when you were a child^?**  **(^under 13 years old)** | **None *** ‘No passive smoking’;  **Not under 13** ‘Passive smoking but no  exposure under 13’;  **Yes under 13** ‘Passive smoking with  exposure under 13’ |
| **Physical activity** | **(1) How many times did you do each of these activities (walking continuously for at least 10 minutes, moderate or vigorous physical activity) last week? and**  **(2) If you add up all the time you spent doing each activity last week, how much time did you spend altogether doing each type of activity?**  *Each minute of walking or moderate intensity activity was counted as 1 minute and each minute of vigorous intensity activity as 2 minutes.*  *Total time spent for all activities was calculated.* | **‘0’*;**  **‘>0 to ≤150’;**  **‘>150 to ≤300’;**  **‘>300’ minutes per week** | | **(1) Just before you, or your partner, became ill, in a NORMAL week, how many times did you engage in VIGOROUS exercise lasting for 20 minutes or more?**  **(2) Just before you, or your partner, became ill, in a NORMAL week, how many times did you engage in LESS VIGOROUS exercise lasting 20 minutes or more?** | **(please refer to the table below ^)**  **‘Nil to very low activity’ *;**  **‘Low activity’;**  **‘Moderate activity’;**  **‘High activity’** |
|  |  |  |  | **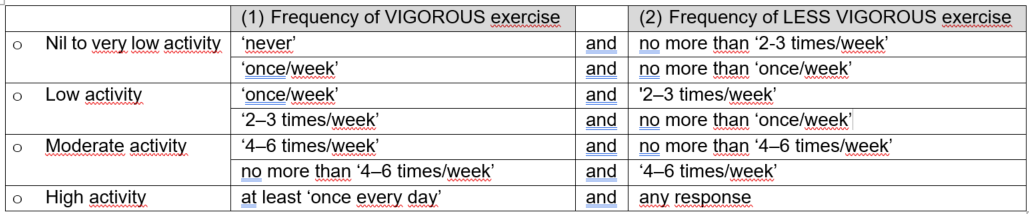** | |
| **Dietary fruit intake** | **About how many serves of (fruit or glasses of fruit juice) do you usually have each day?** | **‘≥0 to <1’ *;**  **‘≥1 to <2’;**  **‘≥2’ serves per day** | | **Five to ten years ago, in the evenings, about how many times a week would you eat the following (fruit)?** | **‘≥0 to <7’ *;**  **‘≥7’ times per week** |
| **Dietary vegetable intake** | **About how many serves of (cooked or raw) vegetables do you usually eat each day?** | **‘≥0 to <3’ *;**  **‘≥3 to <5’;**  **‘≥5’ serves per day** | | **Five to ten years ago, in the evenings, about how many times a week would you eat the following (vegetable)?** | **‘≥0 to <7’ *;**  **‘≥7’ times per week** |
| **Dietary intake of red meat** | **(1) About how many times each week do you eat (beef, lamb or pork)? and**  **(2) Please put a cross in the box if you NEVER eat: [red meat, pork ham, any meat]** | **‘Never’ *;**  **‘>0 to ≤2’;**  **‘>2 to ≤5’;**  **‘>5’ times per week** | | **“Five to ten years ago, in the evenings, about how many times a week would you eat the following (beef/lamb)?”** | **‘Never’ *;**  **‘>0 to ≤2’;**  **‘>2 to ≤5’;**  **‘>5’ times per week** |
| **Self-reported history or recent treatment of Diabetes** | **(1) Has a doctor EVER told you that you have: diabetes? or**  **(2) Have you taken any medications, vitamins or supplements for most of the last 4 weeks? [Diabex, Diaformin, Metformin]** | **‘No’ *;**  **‘Yes’** | | **(1) Has a doctor EVER told you that you had (Pre-diabetes/Diabetes)? or**  **(2) Before you, or your partner, became ill, were you ever treated for: Diabetes?”** | **‘No’ *;**  **‘Yes’** |
| **Use of oral contraceptives^§^** | **(1) Have you ever used the pill or other hormonal contraceptives?**  **and**  **(2) If Yes, for how long altogether have you used hormonal contraceptives?** | **‘Never’ *;**  **‘Yes and <5 years’;**  **‘Yes and ≥5 years’** | | **“Have you ever used the pill or other hormonal contraceptives?”** | **‘No’ *;**  **‘Yes’** |
| **Use of menopausal hormone therapy^§^** | **(1) Have you ever used hormone replacement therapy (HRT)?**  **and**  **(2) Are you currently taking HRT?** | **‘Never user’ *:**  **‘No’ to (1) & (2)**  **‘Former user’:**  **‘Yes’ to (1) & ‘No’ to (2)**  **‘Current user’:**  **‘Yes’ to (1) & (2)** | | **(1) Have you ever used hormone replacement therapy (HRT)?**  **and**  **(2) Were you taking HRT when you, or your partner, became ill?** |  |
| **Variables** | **Questionnaire items for the 45 and Up Study only** | | | | **Categorical grouping** |
| **Self-reported history or recent treatment of chronic disease** | **Has a doctor EVER told you that you have heart disease or high blood pressure or stroke? or**  **In the last month have you been treated for heart attack/angina or other heart disease or high blood pressure or high blood cholesterol?** | | | | **‘No’ *;**  **‘Yes’** |
| **Chronic obstructive pulmonary disease (COPD)** | **A record in the APDC (from July 2001 to recruitment date) with a diagnosis code for bronchitis, chronic bronchitis, emphysema or other COPD (i.e., ICD10-AM J40-J44) or a PBS record (from June 2004 to recruitment date) where a prescription for tiotropium bromide monohydrate (mainstay treatment for COPD) was filled.** | | | | **‘No’ *;**  **‘Yes’** |
| **Self-reported Use of anti-hypertensive medications** | **“Have you taken any medications, vitamins or supplements for most of the last 4 weeks?”**  **(1) Angiotensin converting enzyme inhibitor (ACEI): Captopril (Capoten), Enalapril (Renitec), Lisinopril (Prinivil, Zestril), Fusinopril (Monopril), Quinapril (Accupril), Ramipril (Tritace), Perindopril (Coversyl, Coversyl plus);**  **(2) Angiotensin receptor blocker (ARB): Irbesartan (Avapro, Karvea), Candesartan (Atacand), Losartan (Cozaar), Telmisartan (Micardis), Valsartan (Diovan);**  **(3) Calcium channel blocker (CCB): Diltiazem (Cardizem, Vasocardol), Amlodipine (Norvasc), Nifedipine (Adalat), Felodipine (Plendil).** | | | | **None ***: ‘No use of any of (1), (2) or 3)’*;  **ACEI**: ‘Use of (1) ACEI only’;  **ARB**: ‘Use of (2) ARB only’;  **CCB**: ‘Use of (3) CCB only’;  **≥2 of 3:** ‘Use of two or three of (1), (2) or (3)’ |
| **Dietary intake of processed meat** | **(1) “About how many times each week do you eat (processed meat)?” and (2) “Please put a cross in the box if you NEVER eat: any meat.”** | | | | **‘Never’ *;**  **‘>0 to ≤1’;**  **‘>1 to ≤2’;**  **‘>2’ times per week** |
| **Menopausal age^§^** | **(1) “Have you been through menopause?” and**  **(2) “Yes – How old were you when you went through menopause?”** | | | | **‘No’ *,**  **‘Yes’ < 45 years’*;**  **‘Yes ≥45 to <50 years’;**  **‘≥50 years’** |
| * Reference group. ^§^  For women only.  ^Ω^ Based on the “Australian Bureau of Statistics”: Standard Australian Classification of Countries, 2016.  ^ Based on Nunez C, *et al.* Obesity, physical activity and cancer risks: Results from the Cancer, Lifestyle and Evaluation of Risk Study (CLEAR). Cancer Epidemiol 2017;47:56-63. | | | | | |

**Supplementary Table 2. List of countries for the exposure ‘Asian countries of birth’***

| **Major groups** | **Minor groups** | **Countries** |  |
| --- | --- | --- | --- |
| South-East Asia | Mainland SEA | Myanmar, Cambodia, Laos, Thailand, Vietnam. |  |
| (SEA) | Maritime SEA | Brunei Darussalam, Indonesia, Malaysia, Philippines, Singapore, Timor-Leste. |  |
| North-East Asia | Chinese Asia | China, Hong Kong, Macau, Mongolia, Taiwan. |  |
| (NEA) | Japanese and Koreas | Japan, Korea N, Korea S. |  |
| Southern Asia | Southern Asia | Bangladesh, Bhutan, India, Maldives, Nepal, Pakistan, Sri Lanka. |  |
| * Based on the country codes according to Australian Bureau of Statistics (ABS) Standard Australian Classification of Countries (SACC)  https://www.abs.gov.au/AUSSTATS/abs@.nsf/DetailsPage/1269.0Second%20Edition?OpenDocument | | | |

**Supplementary Table 3. Distribution of lung cancer in never-smoking individuals by sex, Asian countries of birth and histology types, 45 and Up Study**

| **Lung cancer in never-smoking individuals** | **Total no.** | **Men** | **Women** |
| --- | --- | --- | --- |
|  | **n = 226** | **n = 87** | **n = 139** |
| **Asian countries of birth, n (%)** | 20 (8.9) | 11 (12.6) | 9 (6.5) |
| **Non-Asian countries of birth, n (%)** | 206 (91.1) | 76 (87.4) | 130 (93.5) |
|  |  |  |  |
| **Adenocarcinoma, n (%)** | 121 (53.5) | 42 (48.3) | 79 (56.8) |
| **Squamous cell carcinoma, n (%)** | 19 (8.4) | 11 (12.6) | 8 (5.8) |
| **Large cell, n (%)** | 19 (8.4) | 9 (10.3) | 10 (7.2) |
| **Others^§^, n (%)** | 67 (29.7) | 25 (28.8) | 42 (30.2) |
| ^§^This group includes small cell carcinoma, carcinoid and other malignant neoplasms of the lungs. | | | |
|  | | | |

**Supplementary Table 4. Hazard ratios (HRs) and 95% confidence intervals (CIs) of primary incident lung cancer cases among never-smoking individuals with adjustment for age and demographic, health and lifestyle factors in the 45 and Up Study.**

|  | **Lung cancer**  **Yes (%) No (%)** | |  | **Model 1 analysis** | | | | **Model 2 analysis** | | | | **Fully adjusted model** | | | |
| --- | --- | --- | --- | --- | --- | --- | --- | --- | --- | --- | --- | --- | --- | --- | --- |
| **Exposures** | **n = 226** | **n = 132,361** | **p-value** | **HR (95% CI)** | | | **p-value** | **HR (95% CI)** | | | **p-value** | **HR (95% CI)** | | | **p-value** |
| **Mean age (SD), years** | 72.4 (11.0) | 62.1 (11.1) | <0.0001 |  |  |  |  |  |  |  |  |  |  |  |  |
| **Mean Height (SD), cm** | 165.9 (10.3) | 167.5 (10.1) | 0.026 | 1.00 | (0.99, | 1.01) | 0.99 | 1.00 | (0.98, | 1.02) | 0.87 | 1.01 | (0.98, | 1.03) | 0.67 |
| **Sex** |  |  | 0.83 |  |  |  |  |  |  |  |  |  |  |  | 0.88 |
| Male | 87 (38.5) | 51,789 (39.2) |  |  | | |  |  |  |  |  | 1.00 (Reference) | | |  |
| Female | 139 (61.5) | 80,339 (60.8) |  |  |  |  |  |  |  |  |  | 1.03 | (0.67, | 1.60) |  |
| **Country of birth** |  |  | 0.0063 |  |  |  | 0.0001 |  |  |  | 0.0004 |  |  |  | 0.0002 |
| Non-Asian | 206 (91.1) | 125,632 (95.1) |  | 1.00 (Reference) | | |  | 1.00 (Reference) | | |  | 1.00 (Reference) | | |  |
| Asian | 20 (8.9) | 6,496 (4.9) |  | 2.44 | (1.54, | 3.87) |  | 2.37 | (1.47, | 3.82) |  | 2.83 | (1.64, | 4.89) |  |
| **Passive smoking (hours/week)** |  |  | 0.34 |  |  |  | 0.39 |  |  |  | 0.43 |  |  |  | 0.37 |
| None | 141 (62.4) | 85,720 (64.9) |  | 1.00 (Reference) | | |  | 1.00 (Reference) | | |  | 1.00 (Reference) | | |  |
| ≤3.5 | 32 (14.2) | 21,738 (16.4) |  | 1.15 | (0.78, | 1.69) |  | 1.15 | (0.78, | 1.70) |  | 1.12 | (0.75, | 1.68) |  |
| >3.5 | 11 (4.9) | 10,375 (7.9) |  | 0.71 | (0.39, | 1.32) |  | 0.73 | (0.40, | 1.36) |  | 0.67 | (0.35, | 1.28) |  |
| Missing | 42 (18.6) | 14,295 (10.8) |  |  |  |  |  |  |  |  |  |  |  |  |  |
| **Physical activity (min/week)** |  |  | 0.19 |  |  |  | 0.67 |  |  |  | 0.65 |  |  |  | 0.49 |
| Nil | 17 (7.5) | 6,711 (5.1) |  | 1.00 (Reference) | | |  | 1.00 (Reference) | | |  | 1.00 (Reference) | | |  |
| 0 to150 | 38 (16.8) | 21,370 (16.2) |  | 0.79 | (0.45, | 1.41) |  | 0.79 | (0.44, | 1.40) |  | 1.06 | (0.53, | 2.12) |  |
| 150 to 300 | 28 (12.4) | 20,935 (15.8) |  | 0.72 | (0.39, | 1.33) |  | 0.71 | (0.39, | 1.31) |  | 0.75 | (0.35, | 1.59) |  |
| ≥300 | 128 (56.6) | 79,488 (60.2) |  | 0.88 | (0.53, | 1.48) |  | 0.87 | (0.52, | 1.47) |  | 1.10 | (0.58, | 2.10) |  |
| Missing | 15 (6.6) | 3,624 (2.7) |  |  |  |  |  |  |  |  |  |  |  |  |  |
| **BMI (kg/m^2^)** |  |  | 0.0006 |  |  |  | 0.048 |  |  |  | 0.048 |  |  |  | 0.052 |
| < 18.5 | 37 (16.4) | 11,917 (9.0) |  | 1.55 | (1.05, | 2.28) |  | 1.56 | (1.06, | 2.31) |  | 1.98 | (1.16, | 3.39) |  |
| ≥ 18.5 - < 25 | 85 (37.6) | 47,744 (36.1) |  | 1.00 (Reference) | | |  | 1.00 (Reference) | | |  | 1.00 (Reference) | | |  |
| ≥ 25 - < 30 | 72 (31.9) | 47,224 (35.7) |  | 0.91 | (0.66, | 1.25) |  | 0.91 | (0.67, | 1.26) |  | 0.92 | (0.65, | 1.30) |  |
| ≥ 30 | 32 (14.2) | 25,243 (19.1) |  | 0.89 | (0.59, | 1.34) |  | 0.90 | (0.60, | 1.36) |  | 1.01 | (0.64, | 1.59) |  |
| **Alcohol consumption (drinks/week)** |  |  | 0.026 |  |  |  | 0.89 |  |  |  | 0.88 |  |  |  | 0.94 |
| ≥ 0 - < 1 | 106 (46.3) | 49,383 (37.3) |  | 1.22 | (0.81, | 1.84) |  | 1.23 | (0.82, | 1.85) |  | 1.07 | (0.67, | 1.70) |  |
| ≥ 1 - ≤ 3.5 | 30 (13.1) | 22,992 (17.4) |  | 1.00 (Reference) | | |  | 1.00 (Reference) | | |  | 1.00 (Reference) | | |  |
| > 3.5 - ≤ 7 | 40 (17.5) | 26,336 (19.9) |  | 1.07 | (0.67, | 1.72) |  | 1.07 | (0.67, | 1.72) |  | 0.94 | (0.54, | 1.63) |  |
| >7 | 45 (19.7) | 30,601 (23.1) |  | 1.14 | (0.72, | 1.82) |  | 1.13 | (0.70, | 1.80) |  | 1.16 | (0.69, | 1.96) |  |
| Missing | 8 (3.5) | 3,049 (2.3) |  |  |  |  |  |  |  |  |  |  |  |  |  |
| **Fruit intake (serves/day)** |  |  | 0.076 |  |  |  | 0.31 |  |  |  | 0.31 |  |  |  | 0.59 |
| ≥ 0 - < 1 | 7 (3.1) | 6,585 (5.0) |  | 1.00 (Reference) | | |  | 1.00 (Reference) | | |  | 1.00 (Reference) | | |  |
| ≥ 1 - < 2 | 55 (24.3) | 40,454 (30.6) |  | 1.06 | (0.48, | 2.34) |  | 1.06 | (0.48, | 2.34) |  | 0.99 | (0.42, | 2.33) |  |
| ≥ 2 | 146 (64.6) | 79,424 (60.1) |  | 1.33 | (0.62, | 2.85) |  | 1.33 | (0.62, | 2.86) |  | 1.19 | (0.52, | 2.73) |  |
| Missing | 18 (8.0) | 5,665 (4.3) |  |  |  |  |  |  |  |  |  |  |  |  |  |
| **Vegetable intake (serves/day)** |  |  | 0.25 |  |  |  | 0.37 |  |  |  | 0.39 |  |  |  | 0.45 |
| ≥ 0 - < 3 | 47 (20.8) | 36,024 (27.3) |  | 1.00 (Reference) | | |  | 1.00 (Reference) | | |  | 1.00 (Reference) | | |  |
| ≥ 3 - < 5 | 63 (27.9) | 37,271 (28.2) |  | 1.31 | (0.90, | 1.92) |  | 1.31 | (0.89, | 1.92) |  | 1.29 | (0.85, | 1.97) |  |
| ≥ 5 | 72 (31.9) | 41,133 (31.1) |  | 1.18 | (0.81, | 1.70) |  | 1.18 | (0.81, | 1.72) |  | 1.08 | (0.70, | 1.67) |  |
| Missing | 44 (19.5) | 17,700 (13.4) |  |  |  |  |  |  |  |  |  |  |  |  |  |
| **Red meat intake (times/wk)** |  |  | 0.045 |  |  |  | 0.25 |  |  |  | 0.28 |  |  |  | 0.57 |
| Never | 31 (13.7) | 14,373 (10.9) |  | 1.00 (Reference) | | |  | 1.00 (Reference) | | |  | 1.00 (Reference) | | |  |
| > 0 - ≤ 2 | 52 (23.0) | 35,869 (27.2) |  | 0.74 | (0.47, | 1.15) |  | 0.75 | (0.48, | 1.17) |  | 0.89 | (0.52, | 1.50) |  |
| > 2 - ≤ 5 | 102 (45.1) | 64,977 (49.2) |  | 0.78 | (0.52, | 1.16) |  | 0.79 | (0.53, | 1.18) |  | 0.94 | (0.58, | 1.54) |  |
| > 5 | 31 (13.7) | 12,606 (9.5) |  | 1.06 | (0.64, | 1.74) |  | 1.08 | (0.65, | 1.78) |  | 1.26 | (0.69, | 2.27) |  |
| Missing | 10 (4.4) | 4,303 (3.3) |  |  |  |  |  |  |  |  |  |  |  |  |  |
| **Family history of LC** |  |  | 0.37 |  |  |  | 0.47 |  |  |  | 0.46 |  |  |  | 0.39 |
| No | 200 (88.5) | 119,255 (90.3) |  | 1.00 (Reference) | | |  | 1.00 (Reference) | | |  | 1.00 (Reference) | | |  |
| Yes | 26 (11.5) | 12,873 (9.7) |  | 1.16 | (0.77, | 1.75) |  | 1.17 | (0.78, | 1.76) |  | 1.22 | (0.78, | 1.93) |  |
| **Asthma requiring treatment** |  |  | 0.079 |  |  |  | 0.16 |  |  |  | 0.17 |  |  |  | 0.33 |
| No | 211 (93.4) | 126,486 (95.7) |  | 1.00 (Reference) | | |  | 1.00 (Reference) | | |  | 1.00 (Reference) | | |  |
| Yes | 15 (6.6) | 5,642 (4.3) |  | 1.45 | (0.86, | 2.45) |  | 1.44 | (0.85, | 2.44) |  | 1.35 | (0.73, | 2.50) |  |
| **Diabetes** |  |  | 0.48 |  |  |  | 0.84 |  |  |  | 0.83 |  |  |  | 0.87 |
| No | 205 (90.7) | 121,539 (92.0) |  | 1.00 (Reference) | | |  | 1.00 (Reference) | | |  | 1.00 (Reference) | | |  |
| Yes | 21 (9.3) | 10,586 (8.0) |  | 0.87 | (0.56, | 1.37) |  | 0.87 | (0.55, | 1.36) |  | 0.87 | (0.52, | 1.46) |  |
| **Processed meat intake (times/week)** |  |  | 0.085 |  |  |  | 0.13 |  |  |  | 0.16 |  |  |  | 0.12 |
| Never | 59 (26.1) | 30,430 (23.0) |  | 1.00 (Reference) | | |  | 1.00 (Reference) | | |  | 1.00 (Reference) | | |  |
| > 0 - ≤ 1 | 54 (23.9) | 43,752 (33.1) |  | 0.66 | (0.46, | 0.95) |  | 0.67 | (0.46, | 0.97) |  | 0.61 | (0.39, | 0.96) |  |
| > 1 - ≤ 2 | 34 (15.0) | 20,275 (15.4) |  | 0.89 | (0.58, | 1.35) |  | 0.91 | (0.59, | 1.39) |  | 0.99 | (0.60, | 1.62) |  |
| > 2 | 24 (10.6) | 18,071 (13.7) |  | 0.70 | (0.44, | 1.13) |  | 0.72 | (0.45, | 1.17) |  | 0.88 | (0.51, | 1.50) |  |
| Missing | 55 (24.3) | 19,600 (14.8) |  |  |  |  |  |  |  |  |  |  |  |  |  |
| **COPD** |  |  | <0.0001 |  |  |  | 0.050 |  |  |  | 0.054 |  |  |  | 0.20 |
| No | 215 (95.1) | 130,159 (98.5) |  | 1.00 (Reference) | | |  | 1.00 (Reference) | | |  | 1.00 (Reference) | | |  |
| Yes | 11 (4.9) | 1,969 (1.5) |  | 1.84 | (1.00, | 3.39) |  | 1.83 | (0.99, | 3.37) |  | 1.63 | (0.77, | 3.44) |  |
| **Anti-hypertensive medication**  **(ACEI, ARB, CCB)** |  |  | 0.0018 |  |  |  | 0.97 |  |  |  | 0.97 |  |  |  | 0.92 |
| Nil | 156 (69.0) | 105,344 (79.7) |  | 1.00 (Reference) | | |  | 1.00 (Reference) | | |  | 1.00 (Reference) | | |  |
| Only ACEI | 24 (10.6) | 9,663 (7.3) |  | 1.09 | (0.71, | 1.68) |  | 1.09 | (0.71, | 1.69) |  | 1.24 | (0.77, | 2.01) |  |
| Only ARB | 25 (11.1) | 10,974 (8.3) |  | 1.01 | (0.66, | 1.55) |  | 1.02 | (0.66, | 1.56) |  | 1.06 | (0.65, | 1.73) |  |
| Only CCB | 11 (4.9) | 3,073 (2.3) |  | 1.26 | (0.68, | 2.34) |  | 1.26 | (0.68, | 2.33) |  | 1.36 | (0.68, | 2.70) |  |
| Any 2 or all of them | 10 (4.4) | 3,070 (2.3) |  | 1.23 | (0.64, | 2.33) |  | 1.23 | (0.65, | 2.35) |  | 1.17 | (0.54, | 2.54) |  |
| SD: Standard deviation BMI: Body mass index LC: Lung cancer COPD: Chronic obstructive pulmonary disease  ACEI: Angiotensin converting enzyme inhibitor ARB: Angiotensin receptor blocker CCB: Calcium channel blocker  **Model 1:** HR based on age as the underlying time variable.  **Model 2:** HR based on age as the underlying time variable and adjusted for sex, SES, region of residence and educational level.  **Fully adjusted model:** HR based on age as the underlying time variable and adjusted for sex, SES, region of residence, educational level, height, Asian country of birth, family history of LC, passive smoking,  asthma requiring treatment, COPD, diabetes, physical activity, BMI, alcohol drinks, fruit intake, vegetable intake, red meat intake, processed meat intake, anti-hypertensive medications. | | | | | | | | | | | | | | | |

**Supplementary Table 5. Odds ratios (HRs) and 95% confidence intervals (CIs) of primary incident lung cancer cases among never-smoking individuals with adjustment for age and demographic, health and lifestyle factors in the CLEAR Study.**

|  | **Lung cancer**  **Case (%) Control (%)** | |  | **Model 1 analysis** | | | | **Model 2 analysis** | | | | **Fully adjusted model** | | | |
| --- | --- | --- | --- | --- | --- | --- | --- | --- | --- | --- | --- | --- | --- | --- | --- |
| **Exposures** | **n = 58** | **n = 1,316** | **p-value** | **OR (95% CI)** | | | **p-value** | **OR (95% CI)** | | | **p-value** | **OR (95% CI)** | | | **p-value** |
| **Mean age (SD), years** | 64.2 (10.8) | 59.2 (12.3) | 0.0012 |  |  |  |  |  |  |  |  |  |  |  |  |
| **Mean Height (SD), cm** | 165.2 (11.1) | 168.2 (11.4) | 0.062 | 0.22 | (0.024, | 1.95) | 0.17 | 0.44 | (0.03, | 7.05) | 0.56 | 0.46 | (0.02, | 10.10) | 0.62 |
| **Sex** |  |  | 0.094 |  |  |  |  |  |  |  |  |  |  |  | 0.71 |
| Male | 18 (31.0) | 554 (42.1) |  |  | | |  |  |  |  |  | 1.00 (Reference) | | |  |
| Female | 40 (69.0) | 762 (57.9) |  |  |  |  |  |  |  |  |  | 1.16 | (0.53, | 2.53) |  |
| **Country of birth** |  |  | 0.0088 |  |  |  | 0.0026 |  |  |  | 0.0087 |  |  |  | 0.025 |
| Non-Asian | 52 (89.7) | 1269 (96.4) |  | 1.00 (Reference) | | |  | 1.00 (Reference) | | |  | 1.00 (Reference) | | |  |
| Asian | 6 (10.3) | 47 (3.6) |  | 4.11 | (1.64, | 10.32) |  | 3.68 | (1.39, | 9.72) |  | 3.78 | (1.19, | 12.05) |  |
| **Passive smoking exposure** |  |  | 0.34 |  |  |  | 0.52 |  |  |  | 0.49 |  |  |  | 0.65 |
| None | > 47 | 1145 (87.0) |  | 1.00 (Reference) | | |  | 1.00 (Reference) | | |  | 1.00 (Reference) | | |  |
| Not under 13 | < 5 | 44 (3.3) |  | 2.25 | (0.77, | 6.56) |  | 2.35 | (0.78, | 7.10) |  | 2.28 | (0.61, | 8.57) |  |
| Yes under 13 | < 5 | 91 (6.9) |  | 0.97 | (0.34, | 2.76) |  | 0.90 | (0.31, | 2.59) |  | 1.20 | (0.40, | 3.61) |  |
| Missing | 0 | 36 (2.7) |  |  |  |  |  |  |  |  |  |  |  |  |  |
| **Physical activity (intensity level)** |  |  | 0.48 |  |  |  | 0.75 |  |  |  | 0.80 |  |  |  | 0.48 |
| Nil to very low activity | 27 (46.6) | 513 (39.0) |  | 1.00 (Reference) | | |  | 1.00 (Reference) | | |  | 1.00 (Reference) | | |  |
| Low activity | > 9 | 218 (16.6) |  | 1.13 | (0.56, | 2.28) |  | 1.13 | (0.55, | 2.31) |  | 1.54 | (0.72, | 3.30) |  |
| Moderate activity | 17 (29.3) | 473 (35.9) |  | 0.76 | (0.41, | 1.42) |  | 0.77 | (0.41, | 1.44) |  | 0.84 | (0.41, | 1.71) |  |
| High activity | < 5 | 76 (5.8) |  | 0.51 | (0.12, | 2.19) |  | 0.56 | (0.13, | 2.44) |  | 0.31 | (0.04, | 2.51) |  |
| **BMI before dx (kg/m^2^)** |  |  | 0.68 |  |  |  | 0.67 |  |  |  | 0.78 |  |  |  | 0.86 |
| < 18.5 | 8 (13.8) | 145 (11.0) |  | 1.02 | (0.45, | 2.34) |  | 1.07 | (0.46, | 2.52) |  | 1.58 | (0.18, | 13.76) |  |
| ≥ 18.5 - < 25 | 24 (41.4) | 477 (36.3) |  | 1.00 (Reference) | | |  | 1.00 (Reference) | | |  | 1.00 (Reference) | | |  |
| ≥ 25 - < 30 | 18 (31.0) | 461(35.0) |  | 0.75 | (0.40, | 1.40) |  | 0.82 | (0.43, | 1.55) |  | 0.82 | (0.42, | 1.59) |  |
| ≥ 30 | 8 (13.8) | 233 (17.7) |  | 0.67 | (0.30, | 1.52) |  | 0.70 | (0.31, | 1.61) |  | 0.77 | (0.31, | 1.89) |  |
| **Alcohol consumption (drinks/week)** |  |  | 0.23 |  |  |  | 0.53 |  |  |  | 0.53 |  |  |  | 0.69 |
| ≥ 0 - < 1 | 25 (43.1) | 459 (34.9) |  | 1.29 | (0.59, | 2.84) |  | 1.34 | (0.60, | 2.99) |  | 0.96 | (0.41, | 2.26) |  |
| ≥ 1 - ≤ 3.5 | 9 (15.5) | 241 (18.3) |  | 1.00 (Reference) | | |  | 1.00 (Reference) | | |  | 1.00 (Reference) | | |  |
| > 3.5 - ≤ 7 | 15 (25.9) | 285 (21.7) |  | 1.33 | (0.57, | 3.09) |  | 1.32 | (0.56, | 3.12) |  | 1.11 | (0.45, | 2.75) |  |
| >7 | 8 (13.8) | 318 (24.2) |  | 0.67 | (0.26, | 1.77) |  | 0.68 | (0.25, | 1.81) |  | 0.58 | (0.20, | 1.64) |  |
| Missing | 1 (1.7) | 13 (1.0) |  |  |  |  |  |  |  |  |  |  |  |  |  |
| **Evening fruit intake (times/week)** |  |  | 0.28 |  |  |  | 0.27 |  |  |  | 0.27 |  |  |  | 0.26 |
| ≥ 0 - < 7 | 21 (36.2) | 593 (45.1) |  | 1.00 (Reference) | | |  | 1.00 (Reference) | | |  | 1.00 (Reference) | | |  |
| ≥ 7 | 30 (51.7) | 619 (47.0) |  | 1.24 | (0.70, | 2.20) |  | 1.19 | (0.66, | 2.12) |  | 1.39 | (0.72, | 2.68) |  |
| Missing | 7 (12.7) | 104 (7.9) |  |  |  |  |  |  |  |  |  |  |  |  |  |
| **Evening vegetable intake (times/week)** |  |  | 0.94 |  |  |  | 0.95 |  |  |  | 0.92 |  |  |  | 0.24 |
| ≥ 0 - < 7 | 22 (37.9) | 492 (37.4) |  | 1.00 (Reference) | | |  | 1.00 (Reference) | | |  | 1.00 (Reference) | | |  |
| ≥ 7 | 34 (58.6) | 777 (59.0) |  | 0.92 | (0.53, | 1.59) |  | 0.90 | (0.51, | 1.59) |  | 0.66 | (0.35, | 1.26) |  |
| Missing | 2 (3.5) | 47 (3.6) |  |  |  |  |  |  |  |  |  |  |  |  |  |
| **Evening red meat intake (times/week)** |  |  | 0.14 |  |  |  | 0.32 |  |  |  | 0.19 |  |  |  | 0.13 |
| Never | 1 (1.7) | 24 (1.8) |  | 1.00 (Reference) | | |  | 1.00 (Reference) | | |  | 1.00 (Reference) | | |  |
| > 0 - ≤ 2 | 16 (27.6) | 421 (32.0) |  | 0.81 | (0.10, | 6.45) |  | 0.75 | (0.09, | 6.12) |  | 0.70 | (0.08, | 6.00) |  |
| > 2 - ≤ 5 | 30 (51.7) | 741 (56.3) |  | 0.83 | (0.11, | 6.43) |  | 0.76 | (0.10, | 6.07) |  | 0.80 | (0.10, | 6.64) |  |
| > 5 | 8 (13.8) | 82 (6.2) |  | 1.87 | (0.22, | 15.93) |  | 2.02 | (0.23, | 18.00) |  | 2.41 | (0.26, | 22.77) |  |
| Missing | 3 (5.2) | 48 (3.7) |  |  |  |  |  |  |  |  |  |  |  |  |  |
| **Family history of LC** |  |  | 0.45 |  |  |  | 0.60 |  |  |  | 0.76 |  |  |  | 0.40 |
| No | 50 (86.2) | 1176 (89.4) |  | 1.00 (Reference) | | |  | 1.00 (Reference) | | |  | 1.00 (Reference) | | |  |
| Yes | 8 (13.8) | 140 (10.6) |  | 1.23 | (0.57, | 2.65) |  | 1.13 | (0.52, | 2.47) |  | 1.43 | (0.62, | 3.26) |  |
| **Asthma requiring treatment** |  |  | 0.55 |  |  |  | 0.51 |  |  |  | 0.53 |  |  |  | 0.74 |
| No | 48 (82.8) | 1126 (85.6) |  | 1.00 (Reference) | | |  | 1.00 (Reference) | | |  | 1.00 (Reference) | | |  |
| Yes | 10 (17.2) | 190 (14.4) |  | 1.26 | (0.63, | 2.55) |  | 1.26 | (0.61, | 2.60) |  | 0.85 | (0.34, | 2.16) |  |
| **Diabetes** |  |  | 0.58 |  |  |  | 0.36 |  |  |  | 0.36 |  |  |  | 0.49 |
| No | > 53 | 1197 (91.0) |  | 1.00 (Reference) | | |  | 1.00 (Reference) | | |  | 1.00 (Reference) | | |  |
| Yes | < 5 | 119 (9.0) |  | 0.61 | (0.22, | 1.74) |  | 0.61 | (0.21, | 1.74) |  | 0.64 | (0.18, | 2.27) |  |
| SD: Standard deviation BMI: Body mass index LC: Lung cancer  **Model 1:** OR as adjusted for age only.  **Model 2:** OR as adjusted for age, sex, SES, region of residence and educational level.  **Fully adjusted model:** OR as adjusted for age, sex, SES, region of residence, educational level, height, Asian country of birth, family history of LC, passive smoking, asthma requiring treatment, diabetes, physical activity, BMI,  alcohol drinks, evening fruit intake, evening vegetable intake and evening red meat intake. | | | | | | | | | | | | | | | |
